# Paralysis Case and Contact Spread of Recombinant Vaccine–derived Poliovirus, Spain

**DOI:** 10.3201/eid1411.080517

**Published:** 2008-11

**Authors:** Ana Avellón, Maria Cabrerizo, Teresa de Miguel, Pilar Pérez-Breña, Antonio Tenorio, Jose Luis Pérez, Maria Victoria Martínez de Aragón, Gloria Trallero

**Affiliations:** 1 These authors contributed equally to this article.; Instituto de Salud Carlos III National Centre of Microbiology, Madrid, Spain (A. Avellón, M. Cabrerizo, T. de Miguel, P. Pérez-Breña, A. Tenorio, G. Trallero); Instituto de Salud Carlos III National Centre of Epidemiology, Madrid (M.V. Martínez de Aragón); Son Dureta Hospital, Palma de Mallorca, Spain (J.L. Pérez)

**Keywords:** poliovirus, poliomyelitis, enterovirus, VDPV, letter

**To the Editor:** The World Health Organization Polio Eradication Initiative has reduced to 4 the number of countries with endemic transmission of wild polioviruses and has reported the widespread circulation of polioviruses that have evolved from attenuated vaccine (oral poliovirus vaccine [OPV]), so-called vaccine-derived polioviruses (VDPVs) (*1*,*2*). VDPVs can reportedly replicate in immunocompromised persons, in whom they produce paralysis, and can spread to contacts and produce paralytic polio in immunocompetent but incompletely immunized persons; these viruses can even cause some polio outbreaks in areas with a low level of vaccination coverage (*2*). When VDPVs are detected, a careful follow-up of VDPV cases and contacts is essential if spread is to be avoided. Because point mutations or recombination events have been associated with reversion to neurovirulence transmission and thus a greater probability of outbreaks (*2*), meticulous molecular studies of the detected strains are recommended.

Spain progressively adopted vaccination with OPV for children in 2004. As a consequence, the incidence of vaccine-like poliovirus detection in recently vaccinated children, which was relatively common up to that time (51 isolates in 2003) (*3*), began to decrease (15 isolates in 2004 and none in 2005 except the case described here). In July 2005, a 14-month-old boy from Morocco with residual paralysis and major histocompatibility class II immunodeficiency was reported through the Spanish Acute Flaccid Paralysis Surveillance System. The patient had received 2 OPV doses at birth and at 6 months of age in Morocco; 8 months later, meningoencephalitis developed. The case was immediately considered suspicious and was therefore monitored at least monthly until the boy died. Sampling was conducted, coinciding with his visits to the hospital to receive therapy with immunoglobulin (γ globulin 0.5 g/kg). His contacts were studied, environmental surveillance was conducted, and molecular analysis of all detected viruses was performed. Laboratory methods for virus detection and characterization, including 10 new reverse-transcription–PCRs designed to cover the entire genome, are detailed in the Table.

**Table:** Laboratory methods used for study of vaccine-derived poliovirus case, Spain, 2005*

procedure	Test	Method†	Sample
Sample preparation	Concentration of sewage for detection of enterovirus in the environment	Concentration with negative charge filters (Millipore, Billerica, MA, USA; 0.45 μm) of 20 L of local sewage	E
	RNA purification from samples (before molecular analysis)	MagAttract Virus Mini-Biorobot (QIAGEN GmbH, Hilden, Germany) from 200 μL of stool sample dissolved in water	S
Classic virology techniques	Cellular culture (Biosafety Level 3) for growing PV	LB20 (transgenic mouse), RD (human rhabdomyosarcoma), HEF (human fibroblast), A-549 (ATCC-CCL185)	S, E
	Immunofluorescence of infected cells	Lim-Beyesh-Melnick A-H and RIVM A-G pools	I
	EV neutralization assay	Antibodies (Chemicon, Temecula, CA, USA)	I
Molecular techniques	Molecular EV detection	RT-nested PCR 5′ UTR (*4*)	S, I, E
	Molecular EV quantification	MutaReal EV real-time PCR kit (Immunodiagnostik AG, Bensheim, Germany)	S
	Molecular EV typing	RT-nested PCR in major VP1 region (*5*)	S, I, E
	PV intratypic characterization	Specific vaccine PV RT-PCR (*6*)	S, I, E
	PV genome sequencing fragment 1	1s: TAAAACAGCTCTGGGGTTGTA (2–22) 1as: CACCACCCAAGAAGCGGCC (1023–1041) 1ns: GCTCTGGGGTTGTACCCACTCC (9–30) 1nas:TAACTCTGGGCAATTCAACGA(1001–1021)	S, I
	PV genome sequencing fragment 2†	2s: CATGCTAAACTCCCCAAAC (945–963) 2as: AGGTGCGCAACATGATGG (1882–1910)	S, I
	PV genome sequencing fragment 3†	3s: CAGACAATTACCAGTCTCC (1814–1832) 3as: ATTACTAAAAATGCATTGGTTCCC (2518–2541)	S, I
	PV genome sequencing VP1 fragment†	VP1s: ACAACACACATTAGTCAAGAGGCTA (2449–2473) VP1as: GGATTTGGACACCAAAACAAAGC (3385–3407)	S, I
	PV genome sequencing fragment 4†	4s:GTGCCCACGACCTCCA (3288–3303) 4as: CTTGGGTGCGACATCTCA (4042–4059)	S, I
	PV genome sequencing fragment 5†	5s: TAATCAAAATTATCTCATCACTTGTG (3962–3987) 5as: CATGAGCGAGTACTCCAGA (4872–4889)	S, I
	PV genome sequencing fragment 6†	6s: CTGGCCAGGAGATTCG (4834–4949) 6as: AAATGATGGAGTTTTGATCGT(5725–5747)	S, I
	PV genome sequencing fragment 7†	7s: AGGCAGGAACTAATCTTGAAA (5630–5650) 7as: CTAAGTATGTAGGCAACAAGAT (6164–6185)	S, I
	PV genome sequencing fragment 8†	8s: CAAAAATGATCCCAGGCTCA (6117–6136) 8as: AAACCTACAAGGGCATAGATT (6917–6937)	S, I
	PV genome sequencing fragment 9†	9s: CAGGCACATCAATTTTTAACTC (6857–6878) 9as: GGTAAATTTTTCTTTAATTCGGGG(7416–7439)	S, I
	Additional PV sequencing primers	447as: CCGGCCCCTGAATGCGGC (447–464) 4666s: CCAGACGGAGCAGACATG (4666–4683)	S, I

*E, local sewage; S, stools; I, isolates; EV, enterovirus; PV, poliovirus; UTR, untranslated region; VP1, virus capsid protein. †Sense (s) and antisense (as) primers: 5′ → 3' sequence (position according to X00595). n, nested. All reverse transcription–PCR (RT-PCR) systems had the same conditions: 5 μL of clinical samples (case) or isolates (contacts) were added to the reaction mixture (final volume 50 μL): AMV/Tfl 1X reaction buffer, 2 mmol/L MgSO_4_, 200 μM each dNTP, 1 μM each primer, 5 U of AMV RT, and 5 U of Tfl DNA polymerase (Access RT-PCR System, Promega, Madison, WI, USA). First RT step of 45 min at 48°C, 2 min at 94°C, 45 cycles of denaturation (94°C, 2 min), annealing (53°C, 1 min), and elongation (68°C,1 min 30 s).

Serotype 2 VDPVs were detected in all 10 stool samples of the patient with residual paralysis for 6 months, until he died, and in 3 of the 7 family contacts analyzed (father and 2 brothers, 11 and 13 years of age, none with confirmed previous vaccination). One of the contacts, considered immunocompetent, shed virus for 216 days (5 fecal samples in which 5 complete genomes were obtained and 1 additional fecal sample in which virus capsid protein l [VP1] could be amplified); a stool sample collected on day 284 was negative. Technical problems delayed sewage sampling. When sewage from the area in which the patient and positive contacts lived was sampled on February 8, 2006, no polioviruses were detected; however, an echovirus 30 was detected. Poliovirus viral load fluctuated (10^6^–10^9^ copies/mL in the paralysis-affected person), decreasing after each immunoglobulin therapy dose (Figure 1 in the Technical Appendix). The corresponding level was <10^5^ in the contacts. The highest value of viral load was recorded in the patient’s final sample, taken before he died. Homology of the VP1 gene with respect to the original vaccine PV2 fluctuated from 97.8% to 98.6% in the case samples but remained constant (98.4%) in the contact samples (Figure 1 in the Technical Appendix). All studied polioviruses featured the following nucleotide substitutions in the 5′ untranslated region: G309A, T344C, T355C, T398C, A481G, T500C, and T743C (Figure 2 in the Technical Appendix). Furthermore, the final sample from the patient had A476C, G505T, T588A, and A738C. Several nucleotide substitutions detected in VP1–4 were common to all samples (Figure 2 in the Technical Appendix); 5 resulted in amino acid changes, including T2909C (VP1 I143T) and G3277A (VP1 V266I). All samples contained 2 noncontiguous recombination fragments Sabin 2/Sabin 1 in the nonstructural genes, including the entire 3C gene and the 3′ half of the 3D-pol (Figure 2 in the Technical Appendix) as in other reports (*7*–*10*). Both fragments, when compared with C species enterovirus, were closely related to Sabin 1 (99.6% and 97.9%, respectively). Specific nucleotide and amino acid comparisons among the isolates are detailed in Figure 3 in the Technical Appendix.

According to the proposed classification (*2*), all the detected viruses were iVDPVs (isolated from immunocompromised patients) that spread only to close contacts because they were not detected in local sewage. If we assume that the greater the amount of viral excretion in feces that occurs, the higher number of replicating polioviruses (as well as the potential for greater genetic diversity), the patient had a more active infection (that responded to the therapy) than did the contacts. Fluctuation in homology to the parental OPV strain might be due not only to the calculation method (calculation was made on the basis of the majority-base call at each chromatogram position, and case sequences presented many mixed nucleotide positions) but also to immunotherapy. Treatment appeared to have decreased virus replication, probably by its action mainly on species with greater fitness and higher replication rates (those that were more similar to the original Sabin strain). As a consequence, treatment might produce a bottleneck that unmasked more divergent species. Both the case and contact strains had intertypic Sabin 1/Sabin 2 recombination in nonstructural genes and also shared most of the nucleotide and amino acid substitutions. However, pathologic changes occurred only in the patient whose immunologic mechanisms were affected and whose viral load was consequently much higher. A recent report (*1*) suggests that VDPVs can emerge in any country that uses OPV with insufficient vaccine coverage. In a polio-free IPV-user country, poliomyelitis can arise and spread to contacts who are not properly vaccinated. In the case we present here, the high level of vaccination coverage in Spain and the rapid control of close contacts achieved through the surveillance and control programs prevented virus spread. In the global pre-eradication phase, countries are recommended to change vaccination from OPV to IPV. However, IPV-adopting countries commonly share borders with OPV-adopting countries and residents may travel back and forth; thus, although the probability of VDPV circulation decreases, it does not reduce to zero. Therefore, active surveillance, rapid classification of isolates, and molecular characterization of the virus are essential.

## Supplementary Material

Technical Appendix Paralysis Case and Contact Spread of Recombinant Vaccine-derived Poliovirus, Spain
