# Supplementary material for: Paralysis Case and Contact Spread of Recombinant Vaccine–derived Poliovirus, Spain
Source: Emerg Infect Dis. 2008 Nov;14(11):1807–9. doi: 10.3201/eid1411.080517 (PMC2630745; doi:10.3201/eid1411.080517)
Supplement: Technical Appendix — Paralysis Case and Contact Spread of Recombinant Vaccine-derived Poliovirus, Spain [file 08-0517_Techapp-s1.pdf]

Paralysis Case and Contact Spread of Recombinant Vaccine–derived Poliovirus, Spain

Technical Appendix

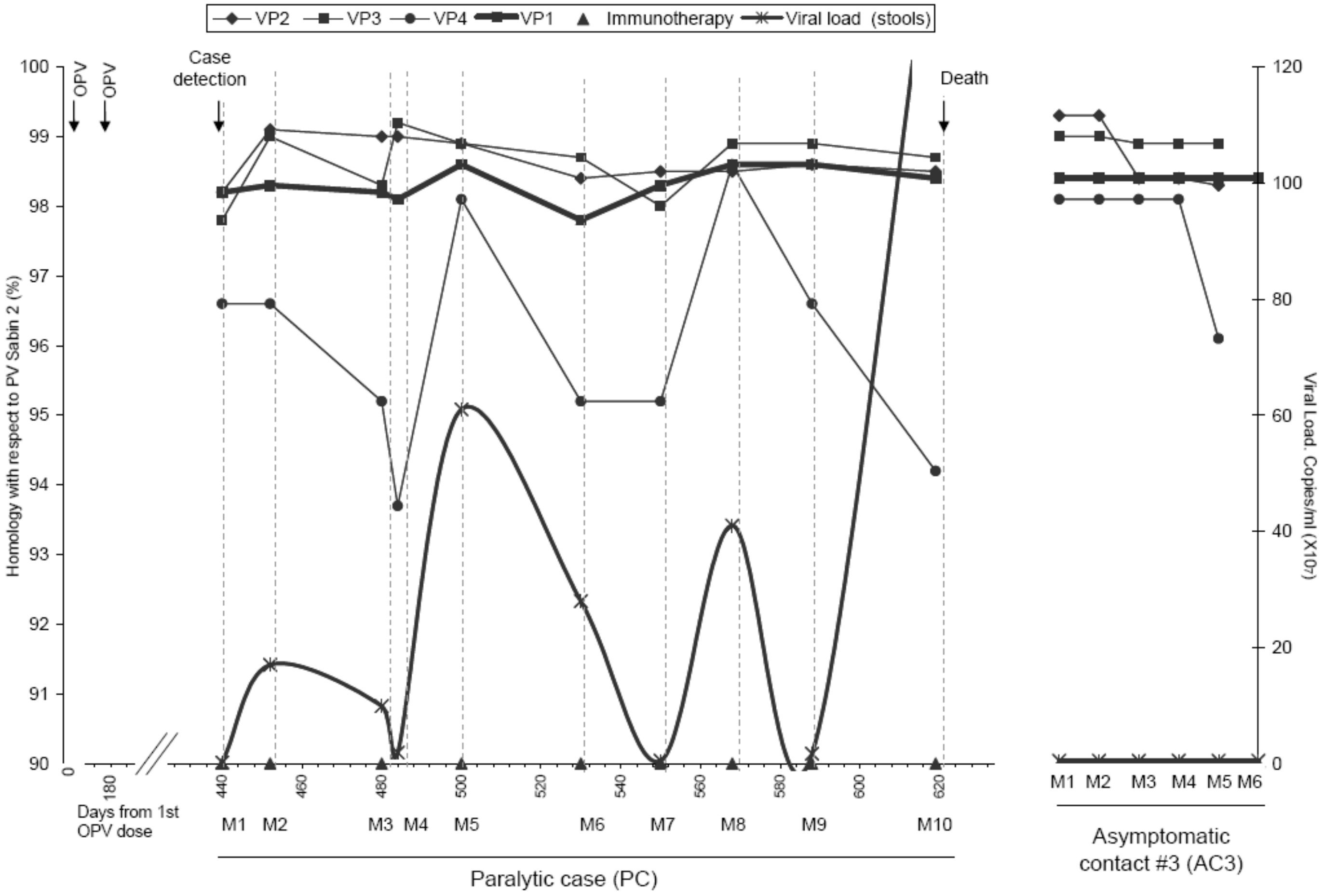

Figure 1. Fluctuation of capsid protein homology to Sabin 2 and viral stool excretion in serial stool samples from the paralysis case-patient (PC, left) and the asymptomatic contact with prolonged excretion (AC3, right). Time of sampling is included in the x-axis; homology percentage with respect to original Sabin 2 strain (X00595) and viral load in stools are in y-axis, left and right, respectively. Oral polivirus (OPV) and immunotherapy doses, case detection, and death of the patient are shown in the graphic.

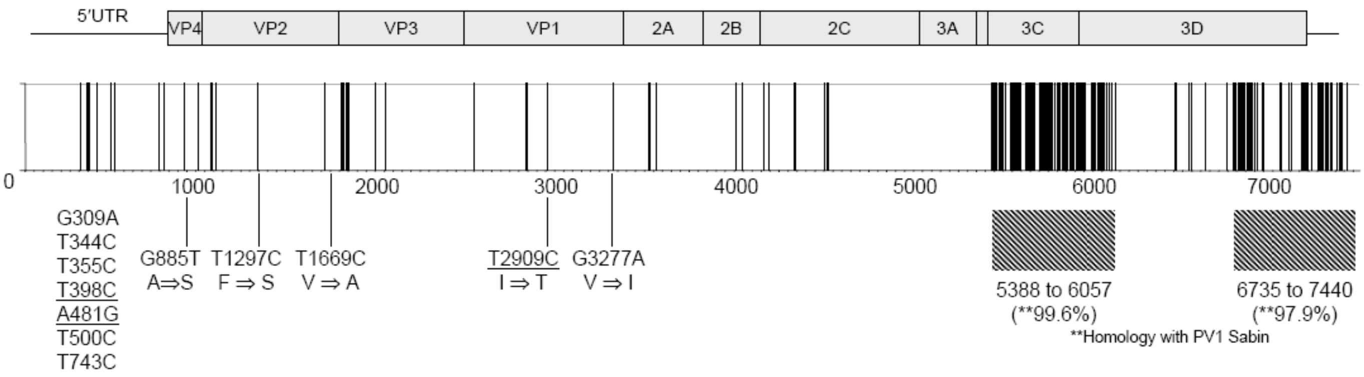

Figure 2. Schematic view of genome alignment of samples with respect to the original Sabin 2 strain (X00595). Nucleotide and amino acid variations common to all samples are marked. Upper lines refer to nucleotide and lower lines to amino acid changes. Underlined substitutions have been previously reported as probably associated with neurovirulence. Sabin 2/Sabin 1 recombination fragments are localized in the genome, and homology with respect to Sabin 1 is detailed in each fragment. Simplot software (http://sray.med.som.jhmi.edu/SCRoftware/simplot) was used to analyze recombination patterns. GenBank sequences described in this work are EU566934–EU566940 for contact samples sequences and EU566941–EU566950 for case sample sequences.

A

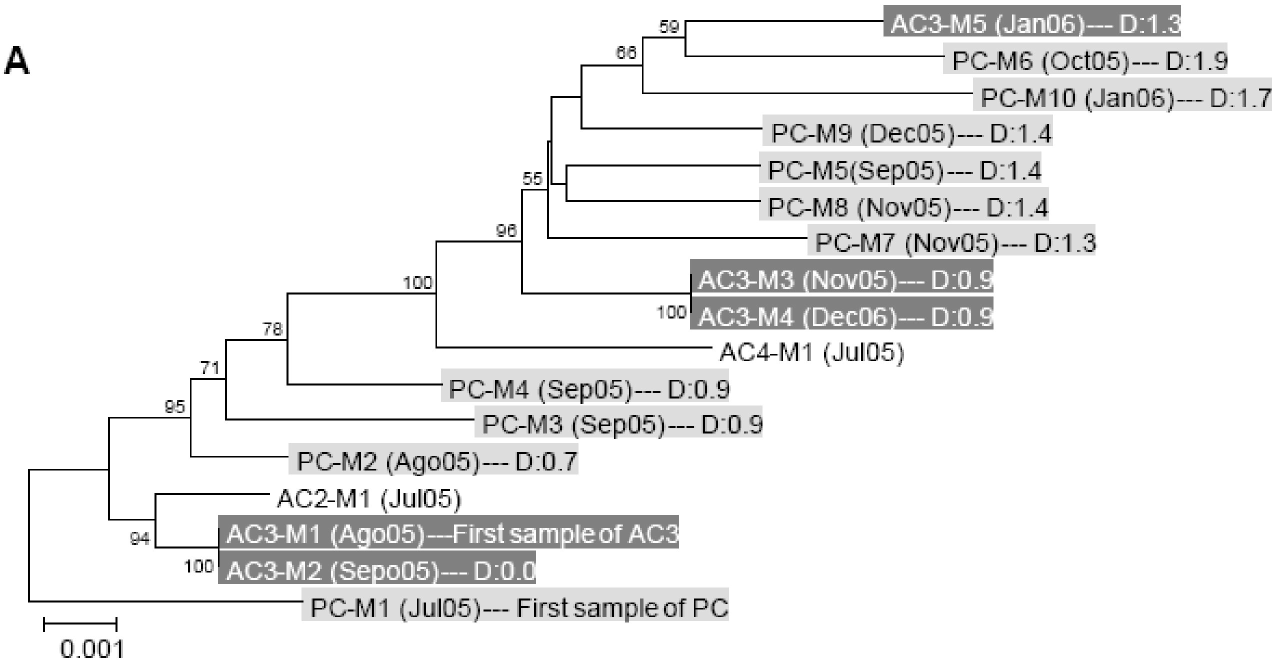

B

		VP2			VP3		VP1		
		NAg3b	NAg2	NAg2	NAg3a	NAg3a and 3b	NAg1	NAg2	NAg3a
	Sabin 2_AY184220	WRK	DTNATNPARN	PRT	NLTSQRK	VELSDTAHSDT	DAPTKRASRLFS	ASTEGD	KDGLT
Paralytic case	PC-M1 (Jul05)	---	-V--------	--I	-------	-----M-----	------------	------	-----
	PC-M2 (Ago05)	---	-A--------	---	-------	-----------	------------	------	-----
	PC-M3 (Sep05)	---	-I--------	---	-------	-----------	------------	------	-----
	PC-M4 (Sep05)	---	-V--------	---	-------	-----------	------------	------	-----
	PC-M5 (Sep05)	---	----------	---	-------	-----------	--------K---	------	-----
	PC-M6 (Oct05)	---	----------	---	-------	-----------	--------K---	------	-----
	PC-M7 (Nov05)	---	----------	---	-------	-----------	--------K---	------	-----
	PC-M8 (Nov05)	---	----------	---	-------	-----------	--------K---	------	-----
	PC-M9 (Dec05)	---	----------	---	-------	-----------	--------K---	------	-----
	PC-M10 (Jan06)	---	----------	---	-------	-----------	--------K---	------	-----
	AC2-M1 (Jul05)	---	-V--------	---	-------	-----M-----	--------K---	------	-----
	AC4-M1 (Jul05)	---	----------	---	-------	-----------	--------K---	------	-----
Asymptomatic contact with prolonged excretion	AC3-M1 (Ago05)	---	-V--------	---	-------	-----M-----	--------K---	------	-----
	AC3-M2 (Sep05)	---	-V--------	---	-------	-----M-----	--------K---	------	-----
	AC3-M3 (Nov05)	---	----------	---	-------	-----------	--------K---	------	-----
	AC3-M4 (Dec05)	---	----------	---	-------	-----------	--------K---	------	-----
	AC3-M5 (Jan06)	---	----------	---	-------	-----------	--------K---	------	-----

Figure 3. A) Nucleotide phylogenetic tree (all bases considered), including case and contact samples. Phylogeny reconstruction method: neighbor-joining. Positions included: 1st+2nd+3rd+noncoding. Substitution model: maximum composite likelihood. Bootstrap (1,000 replicates). Divergence percentage of follow-up sequences with respect to the first isolate in each patient is shown. Divergence calculation was made with MegAlign 6.1 software (DNASTAR package). GenBank sequences described in this work are EU566934–EU566940 for contact samples sequences and EU566941–EU566950 for case sample sequences. B) Detail of predicted neutralizing antigenic sites (estimated according to previously described PV1 and 3) and amino acid changes with respect to the AY184220 Sabin 2 sequence.
